# Chronic recurrent multifocal osteomyelitis in N. Greece: disease burden in pediatric patients

**DOI:** 10.1186/1546-0096-13-S1-P172

**Published:** 2015-09-28

**Authors:** M Trachana, P Pratsidou-Gertsi, E Papadimitriou, A Anastasiou, E Karatza, G Pardalos, E Roilides

**Affiliations:** 1 Pediatric Immunology and Rheumatology Referral Center, Ippokration Hospital, First Department of Pediatrics, Aristotle University, Thessaloniki, Greece; 2 Ippokration General Hospital, Dept of Radiology, Thessaloniki, Greece; 3 Ippokration Hospital, Third Department of Pediatrics, Aristotle University, Thessaloniki, Greece

## Introduction

Chronic Recurrent Multifocal Osteomyelitis (CRMO) is classified among the rare auto-inflammatory diseases. It has a genetic predisposition and a pathogenesis largely under investigation. Greek publications regarding CRMO in pediatric patients are currently absent.

## Objectives

The aim of the study was to describe the disease phenotype, course and outcome of CRMO Northern Greek pediatric patients, and assess the disease activity at diagnosis by the recently proposed Jansson clinical score, which has a cut-off value of 39.

## Patients and methods

Pediatric patients with a diagnosis of CRMO diagnosed and followed-up a tertiary University Hospital during the last decade (2005-2014) were included in this retrospective study.

## Results

Nine patients (female:8, mean age: 10 yrs) were diagnosed with CRMO. The main presenting symptoms in all patients were inflammatory pain in multiple sites, mainly in the metaphyses and epiphyses of long bones in lower limbs (mostly in proximal femur (4/9)) and swelling (7/9). 6/9 patients reported hip pain and only 1, clavicular pain. At diagnosis, fever was noted in 5/9 patients, a raised ESR (mean: 52.5mm/h) in 6/9 and CRP >1mg/dl (≤ 0.5mg/dl) in 5/9. Location of MRI lesions were vertebral (3/9), thoracic rib (1/9), femur (5/9), fibula (5/9) and tibia (3/9). The predominantly affected joints were the hip (5/9) and the ankle (5/9). The calculated clinical score at diagnosis was >39 in 8/9 patients and 36 in 1 pt.

In respect to treatment, the applied NSAIDs provided partial pain relief without preventing the disease course in 8/9 patients; notably, indomethacin had no effect in 1/9 pt. Steroids combined with methotrexate were administered in 5/9 patients, but 4/5 required an anti-TNF to achieve remission. In 2/9 patients antibiotics did not alter the progress of the disease.

## Conclusions

This is the first Greek study that ranks the severity of this rare multi-faceted disease by Jansson clinical score recording a rather severe phenotype, despite the insidious presenting symptoms at onset. As the disease burden was high, half of the patients required biologics for disease taming. Jansson score proved an easy tool that can primary rank the disease severity and identify refractory cases in need for early aggressive treatment.

